# Information management practices in the WHO African Region to support response to the COVID-19 pandemic

**DOI:** 10.1017/S0950268821001242

**Published:** 2021-05-26

**Authors:** Benido Impouma, Tamayi Mlanda, Arish Bukhari, George Sie Williams, Bridget Farham, Caitlin Wolfe, Franck Mboussou, Sara Botero Mesa, Roland Ngom, Theresa Lee, Olivia Keiser

**Affiliations:** 1 World Health Organization, Regional Office for Africa, Brazzaville, Congo; 2 Institute of Global Health, University of Geneva, Geneva, Switzerland; 3 College of Public Health, University of South Florida, Tampa, Florida, USA

**Keywords:** COVID-19, data management

## Abstract

The rapid transmissibility of the severe acute respiratory syndrome-coronavirus-2 causing coronavirus disease-2019, requires timely dissemination of information and public health responses, with all 47 countries of the WHO African Region simultaneously facing significant risk, in contrast to the usual highly localised infectious disease outbreaks. This demanded a different approach to information management and an adaptive information strategy was implemented, focusing on data collection and management, reporting and analysis at the national and regional levels. This approach used frugal innovation, building on tools and technologies that are commonly used, and well understood; as well as developing simple, practical, highly functional and agile solutions that could be rapidly and remotely implemented, and flexible enough to be recalibrated and adapted as required. While the approach was successful in its aim of allowing the WHO Regional Office for Africa (WHO AFRO) to gather surveillance and epidemiological data, several challenges were encountered that affected timeliness and quality of data captured and reported by the member states, showing that strengthening data systems and digital capacity, and encouraging openness and data sharing are an important component of health system strengthening.

The investigation into a cluster of cases of unusual pneumonia by Chinese health authorities in December 2019, in Wuhan City, China, identified a novel coronavirus, severe acute respiratory syndrome- coronavirus-2 (SARS-CoV-2) as the causative agent [1]. Subsequently, the virus spread globally, arriving on the African continent in February 2020 in Algeria and Egypt. As of 31 March 2021, over 3 million confirmed cases and over 75 thousand deaths have been reported in the World Health Organization (WHO) African Region [2].

The unprecedented scale and magnitude of the coronavirus disease-2019 (COVID-19) pandemic simultaneously affecting all 47 WHO member states required reengineering existing emergency information management systems at the WHO Regional Office for Africa (AFRO) to meet the demand of accurately collecting, analysing and disseminating timely information to enhance monitoring of the pandemic and informing public health response actions. WHO Emergency Response Framework requires timely collection and dissemination of health information during epidemics to guide evidence-based operations and support member states in mounting a public health response [3].

An efficient health emergency information management system for responding to the demand for timely and credible information on the COVID-19 pandemic in the African Region needs to be flexible to use with the ability to capture the different modes of data collection and dissemination, while at the same time being interoperable with other platforms in the region, accessible for operational use and sustainable for reference or future use.

Although the African Region is faced with recurrent outbreaks and other health emergencies annually, most of these events are localised and seldomly transcend national boundaries. Health emergency information systems at AFRO were uniquely adapted to respond to these localised events, which required a relatively limited volume of data.

The rapid evolution and simultaneous outbreak of COVID-19 across all 47 member states in the African Region, while at the same time dealing with recurrent infectious disease events, made collecting large volumes of data and the timely delivery of information products complex. The data required for epidemiological characterisation and efficient monitoring of the COVID-19 pandemic ranged across surveillance (aggregated and case-based, contact tracing, alerts or signals), laboratory (testing data), infection prevention and control (health worker data), case management (clinical profile of cases), public health safety measures and socio-demographic metrics. The difficulty of data collection, processing, analysis and dissemination is not only limited to the wide scope and large volumes of data required, but also the different structures and methods of data collection and dissemination adopted by each member state.

This paper discusses the emergency information management system established by AFRO to address the unique challenges presented by the COVID-19 pandemic in the African Region. We describe the types of data collected through the system and the tools used for processing, analysis and dissemination of information on the pandemic. Key results and challenges encountered are discussed, along with the implications for building and sustaining such systems as part of preparedness for and response to future outbreaks in the region.

The guiding principles for AFRO's information management strategy established the need to:

- Build on tools and technologies that are widely adopted for data management during outbreaks and are understood by targeted end-users.
- Propose simple, practical, highly functional and agile solutions that could be rapidly and remotely implemented, and flexible enough for recalibration and adaptation over time.
- Recognise the human resource and financial constraints in the region and the challenge of quickly scaling digital capabilities and technologies.
- Acknowledge the complexity of allocating limited resources towards competing priorities of the response thus creating a preference for low-cost, high-impact information management solutions.
- Acknowledge a race against time to implement complex solutions.

For efficient implementation and delivery of desired outputs, a five-layer emergency information management system (Fig. 1) for COVID-19 was developed and included: (i) A collection layer that collects data shared by member states, (ii) a processing layer that efficiently transforms, merges and organises the data, (iii) a storage layer that facilitates storage and organisation of the data into an easily discoverable structure, (iv) a provisioning layer that makes the data available to data analysts and other consumers while also managing the security and access control concerns and (v) an information dissemination layer that appropriately delivers data and information to end-users who use it as part of normal business processes or to conduct secondary analyses and reporting.

**Fig. 1.**
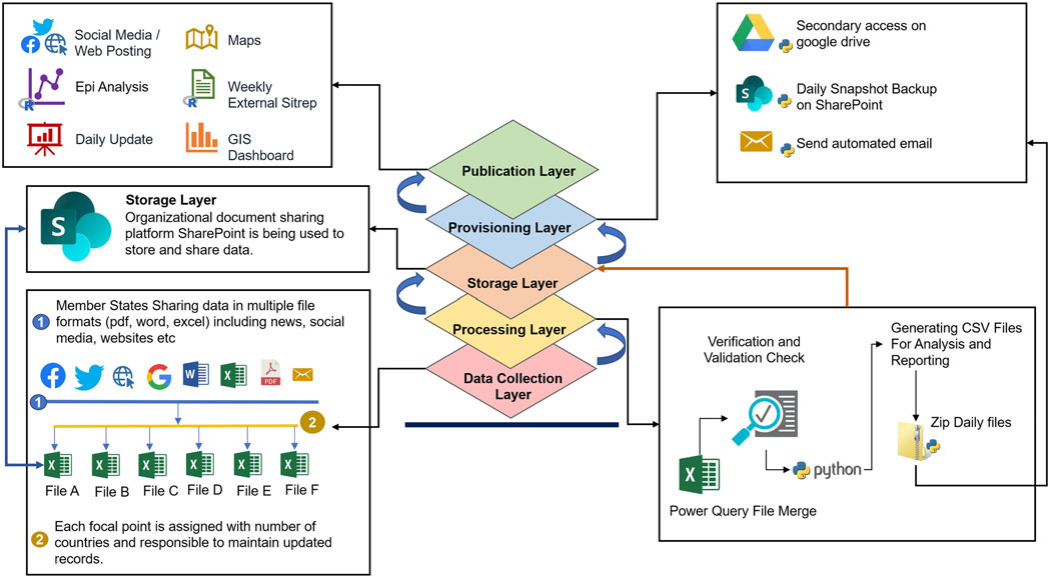
Layers of the COVID-19 information management system.

Member states in the African Region used different tools and platforms for collecting and disseminating the different types of COVID-19 data including different file structures and templates. Data on cases, deaths and other thematic areas of the response were collected at subnational levels using case investigation or reporting forms adapted from the WHO-recommended COVID-19 forms [4]. Each member state defined its mode of data transmission from the peripheral or subnational level to the national level based on existing practices and systems. In some instances, data were captured on Excel-based templates and transmitted via email to the national level, while in others electronic platforms such as Go.Data and the District Health Information Software 2 (DHIS2) [5] were used. These data were collated at national level, analysed and shared with AFRO mainly by electronic mails (emails) in different file structures. File structures shared with AFRO included Excel templates of daily and weekly aggregated data on cases, deaths, recoveries, contacts and laboratory tests; or line list of cases, daily or weekly situation reports in Microsoft Word, Portable Document Format file, or PowerPoint, or image files containing screenshots of the national COVID-19 dashboards. The line lists contained variables related to the socio-demographic, clinical, epidemiological and laboratory data of cases. The situation reports contained information on the epidemiology of the pandemic, public health response measures implemented and challenges encountered.

The main types of data collected by AFRO were case-based or aggregate data on cases and deaths, data on laboratory tests performed, data on contact tracing, as well as signals for monitoring and verifying a unique evolution of the pandemic. Routine health system data, accessible through health management information system portals of the respective Ministries of Health, as well as open-source data on the public health restriction measures available from reputable institutions, were also collected to complement the epidemiological data on COVID-19 reported through official channels.

Official COVID-19 data were sourced daily by a dedicated team of epidemiologists and data scientists from the emails sent to AFRO as well as the official websites or social media pages of the respective member states. The Epidemic Intelligence from Open Sources (EIOS) [6] platform was used for media monitoring COVID-19 signals.

All data captured were entered into an online regional line list using the cloud-based Google spreadsheet solution, leveraging features such as concurrent online editing by multiple users, regular automated and timestamped snapshots, data visualisation and analysis features such as pivot tables, and the ability to export data in common formats such as comma-separated values and Microsoft Excel. An offline data entry process was put in place to address situations of internet outages and mitigate the performance bottlenecks of the collaborative online spreadsheet with many concurrent users. Microsoft Excel was chosen as it featured both an online browser-based editing environment and a feature-rich desktop version capable of handling larger files than would be possible through a web browser on often slow and unstable internet connections. The data were split into multiple files, each with several countries and assigned to specific data entry persons. A SharePoint repository was established to facilitate data synchronisation with the offline editing environments through Microsoft OneDrive. A daily process was established to consolidate the separate Excel files into a single master file and circumvent Excel's row limits. Data verification checks included checks on the numbers of files merged, case reporting dates in each file and consistency of various pivot tables and automated summaries included in each file. A DHIS2 electronic platform was later developed for aggregated data capture and management.

An automated email classification engine was developed to facilitate the classification of all COVID-19-related emails. The C# Microsoft Outlook Add-in employed a heuristic approach using hand-crafted rules and whitelisting [7] to classify incoming emails by country, disease and document type. Rules were coded using compact regular expressions to specify complex text patterns for matching emails by various attributes. Member states were encouraged to adopt a uniform nomenclature for data submissions, which simplified the definition of the pattern matching rules. Once correctly classified, data files were archived onto a centralised document repository from which the data analysis team could easily retrieve them.

Data processing was conducted in various programming languages including R, Python and C#. Data engineering tasks such as merging files, reorganising folders and maintaining repositories and folder locations that contained the latest version of selected document types by country were performed in Python and C#. These tasks were programmed to operate in a semi-automated manner that required human supervision to monitor and perform quality checks.

Data storage was established on an organisation-wide SharePoint environment. All data management was file-based, and SharePoint was used for sharing with both internal and external stakeholders.

Data provisioning was managed through SharePoint, which features robust and granular permissions, and document access control configuration mechanisms. These features were leveraged to allow external consultants and partners to have controlled access to outbreak data and documentation relevant to their work.

All processed data were analysed daily using R and ArcGIS for the entire period of the pandemic by a dedicated team of epidemiologists, data scientists and GIS experts. Data cleaning scripts were built using R packages and functions to identify and merge common variables across all line lists received from the member states, and address inconsistencies in textual formats through conversion, strings matching and manipulation. The variables common to all line lists included date of report, age, sex, place of detection, outcome, dates and results of the tests performed.

Data analysis mainly focused on trends, geo-spatial distribution and epidemiological characterisation of cases by age, sex, disease severity and outcome. Other types of analysis performed included risk profiling of member states based on transmission intensity and response capacity; transmission among special population groups (health workers, students, refugees); building epidemic models for projections and forecasting; testing rates, trends and strategies; and monitoring restriction measures. The proportion of contacts traced was also analysed.

COVID-19 data and information products were disseminated and published through various channels which included:

- Online portal: An interactive ArcGIS Online dashboard [8] that included descriptive epidemiological analyses such as counts and trends of COVID-19 cases and deaths, maps and transmission classification by country.
- Social media: A daily summary of cases and deaths across all member states was prepared and disseminated daily via Facebook and Twitter.
- Mailing-list: An open mailing list was used to disseminate a weekly bulletin specifically covering the COVID-19 pandemic in the region to a wide range of subscribers from the general public, specialised institutions, Ministries of Health, partners and professionals.
- Website: The WHO AFRO website [9] was also used to publish and disseminate various statistics, external situation reports and guidance documents on the outbreak.

Public views of the ArcGIS dashboard as well as social media posts were monitored.

Daily COVID-19 reports or data as of 31 March 2021, were received from a total of 46 member states constituting 98% completeness. Completeness was defined as the proportion of the 47 member states in the African Region that had shared reports on the daily count of cases, deaths and recoveries covering the entire period of the pandemic up till 31 March 2021. A total of 8813 daily situation reports were sent to AFRO as either MS Word, PDF files, or screenshot of dashboards while 1494 were shared using Excel-based templates of aggregated cases, deaths, recoveries and tests. Timeliness of daily reports ranged between 70 and 75% over the period of the pandemic. Timeliness was defined as the proportion of reports for the previous day received at AFRO by 8:00 am Central African Time (CAT) the next day.

A total of 39 (83%) member states shared line lists at least once; with three (6%) consistently sharing at least once a week. On the EIOS platform, out of 64 334 articles related to COVID-19 in the African Region, a total of 844 signals were captured and verified with the member states.

A total of 19 490 emails were automatically tagged by the email classification engine as containing COVID-19 information. Of these 17 205 (88%) contained situation reports and data and were automatically processed and archived onto the document repository.

The interactive ArcGIS online dashboard attracted over 160 million views as of 31 March 2021. A total of 1460 daily social media posts on Facebook and Twitter in both English and French languages were published as of 31 March 2021. Total views ranged from 2 to 5 million people daily. Fifty-two weekly bulletins on the COVID-19 pandemic in the African Region were published. The number of subscribers increased from 3500 at the onset of the pandemic to over 6500 as of 31 March 2020. Thirty-four weekly external situation reports were published on the WHO AFRO website.

These findings showed that despite the enormous challenges presented by the COVID-19 pandemic, the emergency information management system set up at AFRO was able to collect, process and regularly disseminate timely information on the COVID-19 pandemic in the African Region to a wide audience. Adopting a frugal innovation approach [10] by re-purposing features of existing systems and technologies allowed development of a fit-for-purpose information management system without exorbitant costs amidst the complexities posed by the COVID-19 pandemic.

The encompassing nature of the information management system was not only crucial to the collection and processing of high volumes of data, different file structures and formats and different modes of data transmission, but also ensured that epidemiologists and other experts could readily access the data in easy-to-use formats for analysis and production of key information products to inform policy-making and public health responses.

The high completeness of daily reports shows that information products on COVID-19 routinely disseminated by AFRO covered the latest information from almost all the member states in the region and could be a key factor accounting for the growing number of subscribers and views on the different information dissemination platforms.

Despite these efforts, several challenges have been encountered; we highlight the five main ones. First, member states' capacities for data collection varied and the ability to adapt fit-for-purpose and interoperable national information management systems for COVID-19 was hampered by limited resources in several member states. This led to sub-optimal data capture across the different thematic areas of the pandemic, as well as affected the timeliness and quality of data shared by the member states.

Second, some member states frequently changed reporting templates as the pandemic evolved while in some instances nomenclature rules for emailing reports were not followed. This meant that the information management systems at AFRO had to be frequently adapted to these changes, while staff had to carefully work through each file and multiple emails to ensure that no data or reports were missed. Although email was a dominant means for data sharing by the member states, there are inherent limitations in the context of data security and confidentiality.

Third, the contents of situation report templates and variables captured on line listings varied. This posed a challenge for regional aggregation and disaggregation of cases by essential socio-demographic, clinical and epidemiological profiles. For example, ages of cases were reported using different age bands in the situation reports, and clinical severity of cases were not always captured or updated in the line list. Additionally, although the R statistical environment provided a means for line list merging and cleaning, limited and inconsistent standardisation of values required time-consuming review of these files and adjusting cleaning rules.

Fourth, the wide use of spreadsheets made it challenging for the member states to prevent data loss and enforce data security; uniformly implement strict data collection standards; consolidate multiple files and manage data quality concerns. Spreadsheets do not implement field-level access control or encryption, and personally identifiable information (PII) can be easily accessed, further increasing the reluctance of member states to share line list outside their jurisdictions.

Lastly, political barriers to data sharing hindered AFRO's ability to timely and accurately monitor the transmission dynamics of COVID-19 in some settings. For example, since 7 May 2020, the United Republic of Tanzania had not shared data on the COVID-19 pandemic. Other member states established political structures that coordinated COVID-19 data flows but did not adhere to IHR-2015 data-sharing requirements.

These challenges posed limitations for the information management system at AFRO, particularly for characterising the clinical profile of cases in the region since case-based data would be required. Additionally, although reporting completeness is very high, it is unlikely that these reports captured all COVID-19 infections in the region due to low testing rates and limited interoperability of the different data capture systems within the member states.

AFRO has relied on complementary information from reputable open-source data, research studies and partnerships with other institutions to circumvent the challenges. The EIOS platform has been routinely used to monitor signals in areas where official reporting is sub-optimal or hindered. Additionally, AFRO has offered standardised templates and flexible electronic tools for data collection to support member states in the region. One example is the use of Go.Data, an electronic tool for capturing case-based and contact tracing data. These offers included building technical capacities through training of data managers and surveillance officers on the use of these tools.

A key lesson learned is that the ability to use existing technologies requires having the right skills mix and capacities to quickly adapt a fit-for-purpose information management system in a challenging context. The inherent weaknesses in information management for epidemics across the region requires prioritisation for investments. National governments, international partners and institutions should leverage the opportunities provided by the increasing penetration of mobile networks and internet coverage in the region to invest in interoperable hybrid systems with both online and offline features to address the many data collection challenges and streamline reporting from peripheral to national and regional levels.

## Data Availability

The datasets generated during and/or analysed during the current study are available from the corresponding author on reasonable request.
